# Oil Extract of Green Brazilian Propolis, Antioxidant Activity, Safety and Quality Control

**DOI:** 10.3390/molecules31081234

**Published:** 2026-04-08

**Authors:** Murilo Alberici de Oliveira, Giovanna Veronezzi, Guilherme Perez Pinheiro, Marcia Ortiz Mayo Marques, Alexandra Christine Helena Frankland Sawaya

**Affiliations:** 1 Faculty of Pharmaceutical Science, Campinas State University, Campinas 13083-871, SP, Brazil; muriloalb@hotmail.com (M.A.d.O.); gioveronezzi@gmail.com (G.V.); pinheiro.gperez@gmail.com (G.P.P.); 2 Agronomic Institute (IAC), Plant Genetic Resources Center (Phytochemistry), Campinas 13075-630, SP, Brazil; mortiz@iac.sp.gov.br

**Keywords:** green Brazilian propolis, total phenolic content, total flavonoid content, antioxidant activity, UHPLC-MS

## Abstract

Propolis is a resin collected by bees from several plant sources and used by humans for centuries. Its commercial use is usually based on alcoholic extracts, which is a drawback for some applications. Conversely, oil extracts are non-toxic and capable of extracting and dissolving a wide range of less polar compounds. As previous studies showed that oil extracts presented bioactivity similar to ethanolic extracts, a reproducible method for the extraction of green Brazilian propolis was developed and patented. The antimicrobial and cytotoxic activities of the ethanolic and oil extracts of green propolis were compared as well as their ultra-high-performance liquid chromatography with high-resolution mass spectrometry (UHPLC-HRMS) profiles, with similar results. A method was developed to recover propolis bioactive compounds from the oily matrix in order to allow its qualitative and quantitative quality control, according to parameters determined by the Brazilian Ministry of Agriculture, and is presented herein for the first time. The total flavonoid and phenolic contents, antioxidant activity and dry mass are comparable to the ethanolic extract. Therefore, OEP can be recommended for the diverse food supplements and cosmetic products that currently use the ethanolic extract of propolis, without the drawbacks of the presence of alcohol in these formulations.

## 1. Introduction

Propolis is a resin collected by bees from several plant sources and used by humans for centuries. Its chemical composition is complex and is related to the flora of the region, as well as its pharmacological activity. Its commercial use is usually based on alcoholic extracts, often using 70% *v*/*v* grain alcohol, with extraction times ranging from 1 day to 6 months. Despite the widespread use of ethanolic extracts of propolis (EEP), their application is limited by the presence of alcohol. Oil extract of propolis (OEP) represents a promising alternative; however, the lack of standardized analytical methodologies for OEP limits characterization and quality control.

On the other hand, oil extracts are one of the oldest forms of extraction; they are non-toxic and capable of extracting and dissolving a wide range of less polar compounds contained in plant materials [1]. Furthermore, they can be easily incorporated in semi-solid, food and cosmetic products, but have not found wide application in propolis extraction to date. However, in the last decades, several studies of OEP reported encouraging results. Buriol et al. (2009) [2] evaluated OEP obtained by maceration of green Brazilian propolis in canola oil for up to 90 days. The authors concluded that the composition of the oil extract was similar to the traditional ethanol extract, based on ESI-MS fingerprinting. The total phenolic and flavonoid contents of this extract were comparable to those of ethanol extracts [2]. Oil extract of Brazilian propolis was shown to be as effective as the ethanolic extract at inhibiting tumor growth and several identified compounds in fractions from the oil extract of propolis were also found in the ethanolic extracts [3]. A fraction of the same oil extract was tested in vitro against *Candida albicans* strains, and found to contain kaempferide and isosakuranetin, flavonoids commonly found in green Brazilian propolis [4]. Furthermore, this oil extract was shown to present stimulant, anxiolytic and antidepressant effects on the central nervous system and antioxidant activity in rats submitted to stress [5]. Another study compared the effect of several extraction solvents on green Brazilian propolis and concluded that flavonoids are extracted by both ethanol and vegetable oils, but the strongly antioxidant dicaffeoylquinic acids are found in more polar extracts, such as ethanol 30% [6]. All of these studies were performed with Brazilian green propolis, which is also the subject of the present study.

Studies using propolis from other origins also presented interesting results. A sample of Italian propolis was extracted using various solvents (hydroalcoholic, glycolic, glyceric solutions and oil) and all of these extracts showed a quite similar polyphenol composition, as well as a comparable antioxidant activity [7]. A sample of poplar type propolis was extracted with olive oil; phenolic content of these extracts was determined by the Folin–Ciocalteu method, as well as the antioxidant activity by two methods. As expected for Poplar propolis, caffeic acid phenethyl ester (CAPE) was found in high concentrations and the oil extract using the highest concentration of propolis (40%) presented the highest total phenolic content and antioxidant activity [8]. A method for the extraction of green Brazilian propolis was developed and patented. The process of extracting the functional compounds of propolis in vegetable oil, obtaining oily extract and oleogel and their applications in food, pharmaceuticals and cosmetics [9] was initially licensed to a Brazilian apicultural company (ITA BRASIL). Herein, the antimicrobial and cytotoxic activities of the ethanolic and oil extracts of green propolis furnished by this company (ITA BRASIL) were compared as well as their ultra-high-performance liquid chromatography with high-resolution mass spectrometry (UHPLC-HRMS) profiles.

In order to commercialize this innovative product, the quality control standards normally applied to the ethanolic extracts of propolis, must also be applied to its oil extract. Therefore, this study aimed to develop a method to recover propolis bioactive compounds from the oily matrix in order to allow its qualitative and quantitative quality control, according to parameters determined by the Brazilian Ministry of Agriculture for green propolis [10]. Unlike ethanol extracts, the solvent (oil) cannot simply be evaporated using temperature. In sequence, the other quality parameters of propolis (total phenolic and flavonoid contents), as well as its antioxidant potential by the DPPH method, were applied.

The results of the antioxidant and antimicrobial activity, as well as the cytotoxicity tests show that oil can substitute ethanol as an extraction solvent, making the oil extract of propolis (OEP) a safe and effective product that can be easily incorporated in capsules, cosmetic or food products. Furthermore, the method developed in this study permits the quality control of the oil extract using the same parameters as for the ethanol extract, so no new legislation is necessary for OEP.

## 2. Results

### 2.1. Cytotoxicity

Initially, the cytoxicity (focusing on the comparative safety of the oil and ethanol extracts) was evaluated, indicating that both samples present similar activity. According to the standardized method used, the results of the green propolis ethanolic extract presented an IC50 of 5180.0 μg/mL. Therefore, according to the R^2^ of the Hill function, the estimate of the initial dose for toxicity studies would be 5967.4 mg/Kg. The oil extract presented an IC50 equal to 68,014.3 μg/mL. Therefore, the estimate of the initial dose for toxicity studies would be 6633.3 mg/kg. As LD50 > 5000 mg/kg is not considered toxic according to Chemical Hazard Classification and Labeling, both EEP and OEP may be considered safe for oral consumption.

### 2.2. Antimicrobial Activity

The antimicrobial activity of both extracts was also compared. The EEP solvent was evaporated and the concentration based on the dry residue; the concentration of the OEP was based on the estimated dry mass of 20.48% (*m*/*v*), according to our method (see Dry Mass). Both were diluted in ethanol and then serial dilutions in broth were performed; the maximum concentration tested was 5 mg/mL. MIC, MBC and MFC are presented in Table 1. Both ethanol and oil extracts were effective against *Staphylococcus aureus* and *Streptococcus mutans* at concentrations below 1 mg/mL, which can be considered effective, and were also bactericidal at these concentrations. Higher concentrations were necessary against *Streptococcus pyogenes* and *Pseudomonas aeruginosa* but were not always effective.

### 2.3. Calculation of Resin and Wax Contents in Raw Propolis to Propose Theoretical Values Expected in Oily Propolis

An aliquot of 100 g of raw green propolis from the same apiary was extracted with ethanol and found to contain approximately 9 g of wax, 31 g of resin and 59 g of insoluble material. To prepare the oil extract of propolis (OEP) 100 g of raw propolis is added to approximately 300 g of sunflower oil, heated and filtered, according to the patented process. So, for 100 g of raw propolis one could expect to obtain approximately 340 g of oily propolis based on the proportions of resin, wax and insoluble material that we observed. This oil extract would have approximately 40/340 × 100 = 11.7% of resin + wax. These are the theoretical values expected in OEP based on the results for this batch of raw propolis. Therefore, in the extraction method, we expect to obtain results close to theoretical values in terms of dry mass, although individual batches of raw propolis can vary substantially.

### 2.4. Extraction Solvent Selection

In the initial tests with ethanol and methanol, 9.5% *w*/*w* of resin was extracted using ethanol and 4.7% *w*/*w* of resins using methanol. However, the less polar bioactive compounds (such as Artepillin C, *m*/*z* 299) were better extracted by the addition of isopropanol (a less polar solvent than ethanol) (Table S1).

The method for the recovery of propolis bioactive resins from the oily matrix was standardized. First weighing 30 mg of the OEP sample in a capped tube and adding 2 mL of EI; vortexing for 2 min, then placing in an ultrasonic bath for 40 min to allow greater contact between the sample and the solvent. The tube was then kept in a freezer at −15 °C for at least 14 h to precipitate the wax and separate it from the solvent. The solution was centrifuged, put for another 2 s in the freezer, and the clear supernatant was removed with a pipette and placed in a new tube.

### 2.5. Results of Ultra-High Performance Liquid Chromatography with High-Resolution Mass Spectrometry (UHPLC-HRMS) Analysis

**Figure 1:**
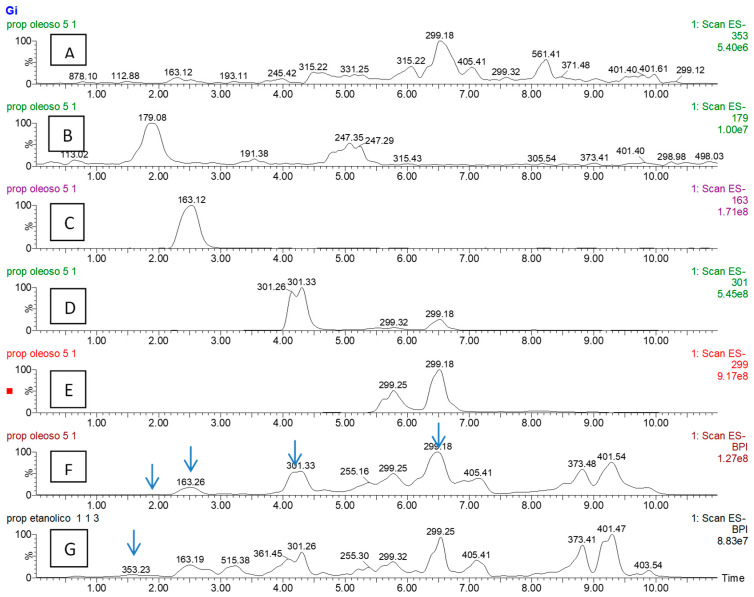
UHPLC-HRMS chromatograms of the extract obtained from OEP with ethanol/isopropanol (95:5). (**A**)—XIC *m*/*z* 353; (**B**)—XIC *m*/*z* 179; (**C**)—XIC *m*/*z* 163; (**D**)—XIC *m*/*z* 301; (**E**)—XIC *m*/*z* 299; (**F**)—TIC OEP and (**G**)—TIC EEP.

Using UHPLC-MS, we were able to compare the ethanol extract with the oil extract and quantify some bioactive compounds (Figure 1). The following compounds: chlorogenic acid, caffeic acid, p-coumaric acid and quercetin were identified and quantified by comparison with calibration curves of their standards. Artepillin-C was identified by a donated isolated fraction but was quantified using the caffeic acid standard curve. It can be seen that the more polar compounds are present in higher concentrations in the ethanol extract (EEP). In contrast, the oil extract extracted a higher concentration of Artepillin-C, an important bioactive ingredient of green propolis (Table 2). However, the overall profile of both extracts (Figure 1F, G) was quite similar.

### 2.6. Total Flavonoid Contents

Total flavonoid contents were initially obtained for batches 2022 and 2023, and the sunflower oil. These were two triplicates of a 30 mg extraction sample from batch 2022 and from batch 2023. Sunflower oil without propolis was also analyzed in the same manner. In these assays, a standard curve for quercetin at concentrations of between 5 and 500 µg/mL was diluted in EI. A blank of the solvents was used. The total flavonoid contents were calculated as 0.55% *m*/*m* for 2022, 0.44% *m*/*m* for 2023 and 0.02% *m*/*m* for sunflower oil (Table 3). For the 2024 sample, the calibration curve was modified (20–300 μg/mL) and read in a 64-well plate instead of individual cuvettes. However, the result of the total flavonoid content was similar (0.45%) to previous batches.

### 2.7. Total Phenolic Contents

To determine the total phenolic content, the Folin–Ciocalteu method and a gallic acid standard curve with concentrations between 1 and 100 μg/mL were used. The total phenolic content was on average, 2.36% *m*/*m* for 2022, 1.70% *m*/*m* for 2023 and 0.17% *m*/*m* for sunflower oil. For batch 2024, the curve was modified, with points 5, 10, 25, 50 and 100 μg/mL of gallic acid, together with the reading of the samples on a 64-well plate. However, the result was similar, 2.05% *m*/*m*, with a greater difference between the previous batches (2022 and 2023) than between them and 2024, showing that these variations in the method did not affect the results (Table 4). It can be observed that the sunflower oil contains some phenolic compounds but contributes very little to the total content of the oil propolis samples.

### 2.8. Antioxidant Activity

Assays using the DPPH method were used to evaluate batches 2022, 2023 and 2024. The ED50, that is, the amount of sample required to obtain 50% antioxidant activity, termed antioxidant potential, was obtained by triplicate readings after 30 min and 1 h of reaction. After one hour, the results were, on average, better (Table 5). Therefore, it is recommended to take the reading after 1 h, when the reaction has stabilized.

### 2.9. Dry Mass

A frequently used method to extract phenolic compounds from edible oils was tested [11] (Márquez-Ruiz et al., 1998), although it was not the first choice due to the use of toxic organic solvents and single-use SEPAK cartridges. This method separates the solution into three phases: non-polar, polar 1, and polar 2. The oil and residual wax were expected to be removed in the non-polar phase (which was discarded), and only polar phases 1 and 2 were weighed. This method was applied to the three batches of OEP (Table S2), but the results (between 41 and 51% of the sample) were much higher than the expected values.

Therefore, using our in-house method: an aliquot of the dry extract was weighed to determine the solids recovered from the oil extract (dry mass). The dry mass of triplicates of the extracts from the three batches (2022, 2023, and 2024) was determined and the results presented in Table 6.

## 3. Discussion

The results of the cytotoxicity assay indicate that both extracts are safe and equivalent. The initial dose estimated for acute oral toxicity testing for both extracts was greater than 5000 mg/kg, so neither are classified in any of the hazardous categories by the United Nations [12] (UN) Globally Harmonized System of Classification and Labeling of Chemicals (GHS). As ethanolic extracts of green propolis have been consumed worldwide for decades, being considered safe, the oil extract, which presented a higher LD50 and is therefore even less toxic, can be consumed safely as well.

The results of the antimicrobial activity are in line with other studies that indicate that green propolis is mainly effective against Gram positive bacteria, such as results below 1 mg/mL for *S. aureus* but over 5 mg/mL *S. pyogenes*. Other studies have presented similar results for EEP with MBC of over 5 mg/mL against *S. aureus*; over 5 mg/mL against S. pyogenes and over 2.5 mg/mL against *S. mutans* [13] (Sawaya et al., 2004). *Candida albicans* also presents a challenge, as EOP was not effective and EEP presented MIC and MFC of 2 mg/mL. However, this result is better than a previous study in which the ethanol extract of green propolis presented a MFC of 20 mg/mL and a MIC of 10 mg/mL [14].

To determine the most adequate solvent to recover the propolis resin from the oil matrix, ethanol and methanol were initially used. We observed that the less polar components of propolis (such as Artepillin C, *m*/*z* 299) were not efficiently extracted with methanol. Ethanol was more effective; however, the addition of 5% isopropanol obtained the highest mass value in the first extraction (therefore better separating the resin from the oil). Solvents containing more isopropanol (50 and 100%) recovered more mass, but this was due to the presence of oil. Small variations were carried out to improve the method and verify if it was robust. Using two subsequent extractions of 1 mL or a single extraction with 2 mL of the same 30 mg, did not significantly modify the result. Furthermore, in the 2024 batch, the centrifugation step was omitted from the extraction because the wax and oil partially re-dissolved in the solvent at room temperature. Since the supernatant remained clear in the freezer, the cloudy material at the bottom of the tube could be discarded. This supernatant solution was used for all the assays (UHPLC-MS analysis, total flavonoid and phenolic contents, antioxidant activity and dry mass).

The direct comparison of ethanolic and oil extracts of green propolis has not been previously reported. Using UHPLC-MS, we were able to compare EEP with EOP and quantify some bioactive compounds (Figure 1). As could be expected, the more polar compounds, such as chlorogenic acid, caffeic acid and p-coumaric acid, are present in higher concentrations in EEP. In contrast, the OEP contained a higher concentration of Artepillin-C, an important bioactive ingredient of green propolis. However, the LC-MS profiles of both extracts were quite similar, indicating that both EEP and EOP contain important bioactive molecules, which was corroborated by the results of their antimicrobial activity.

The total flavonoid contents were calculated as 0.55% *m*/*m* for 2022, 0.44% *m*/*m* for 2023 and 0.02% m/m for sunflower oil and 0.45% for the 2024 batch. The slight changes in the quercetin standard curve calibration curve, as well as reading in cuvettes vs. 64-wellplates, did not significantly modify the results (Table 3). Although sunflower oil contains some flavonoids it contributes very little to the total flavonoid content of the oil propolis samples. Furthermore, all three batches of EOP were above the minimum required by the Brazilian Ministry of Agriculture [10], i.e., 0.25%, and therefore would be acceptable by this parameter, without modifying the existing norm.

The total phenolic contents were calculated a 2.36% *m*/*m* for 2022, 1.70% *m*/*m* for 2023 and 0.17% *m*/*m* for sunflower oil and 2.05% *m*/*m* for the 2024 batch. The slight changes in the calibration curve, as well as reading in cuvettes vs. 64-wellplates, did not significantly modify the results (Table 4). Again, sunflower oil contains some phenolic compounds, but contributes very little to the total phenolic content of the EOP. Furthermore, all three batches were far above the minimum required by the Brazilian Ministry of Agriculture [10], i.e., 0.50%, and therefore would be acceptable by this parameter.

DPPH tests on batches 2022, 2023 and 2024 were performed and the ED50, that is, the amount of sample required to obtain 50% antioxidant activity, which was termed antioxidant potential, was 25 μg/mL on average (Table 5). These results are in line with a recent study [15] that determined the antioxidant capacity by DPPH of twelve green propolis samples, finding a range between 33.86 and 201.29 µg/mL of the extracts. As the lower the ED50, the higher the activity, the result of the oil extracts were superior to the ethanolic extracts reported in that study.

A method frequently used to extract phenolic compounds from edible oils was tested [11], although it was not the first choice due to the use of toxic organic solvents and non-reusable SEPAK cartridges. This method was applied to the three batches of OEP (Table S2), but the results (between 41 and 51% of the sample) were also inconsistent with the expected values of 11.7% (see Section 2.3). Our in-house method (Table 6) reported a dry mass of approximately 20% (SD < 4%), which is above the minimum required by the Brazilian Ministry of Agriculture, i.e., 11%, [10] and therefore would be acceptable by this parameter, without needing to modify the existing norms. However, this value can vary significantly between batches of raw propolis. Further studies with a larger number of batches in the future, could better define this parameter for OEP.

The results reported herein corroborate previous studies [2,3,4,5,6] of the composition of the oil extract of green Brazilian propolis, indicating that its composition and activity are similar to the ethanol extract, although the more polar components (such as chlorogenic acid, coumaric and caffeic acids) are not as well extracted in oil, as shown in Table 2. However, this is the first time that a method is presented to reproducibly recover these resins from the oily matrix, making the quality control of OEP possible using the same standardized methods used for EEP.

## 4. Materials and Methods

### 4.1. Material

Raw green propolis supplied by the ITA Brasil apiary in Bom Jesus—Itapecirica, MG, (20°28′27.3″ S 45°06′31.4″ W) Brazil, in 2022 was analyzed, as well as three batches of OEP produced from green propolis at the same apiary in 2022, 2023 and 2024. A batch of ethanolic extract of green produced in 2022 by the ITA Brasil apiary was also used, for comparative purposes, in some of the studies. In Minas Gerais, Brazil, the standard botanical source of green propolis is *Baccharis dracunculifolia*. Raw propolis is harvested using smart collectors (plastic/metal screens) placed over the walls, crevices and frames, at the end of the summer rains, in the beginning of autumn each year.

### 4.2. Methods to Determine Dry Residue, Wax, and Insoluble Contents in Ethanol Extraction of Raw Propolis

To determine the percentage of resin in the raw propolis sample, three 1 g samples of raw green propolis from the same apiary were weighed. The maceration of 1 g of sample was standardized in 10 mL of ethanol. This mixture was placed in an ultrasonic bath for 30 min to facilitate sample dissolution.

The first filtration through previously weighed paper separated the insoluble components, which are generally composed of soil, plant fragments, and insects. This filtration was performed at room temperature and the solute is stored in a freezer. The paper was dried at room temperature and weighed (insoluble content), discounting the initial mass of the paper.

After storing overnight in a freezer (−20 °C), the solution was filtered again through previously weighed paper to remove the wax that precipitates at low temperatures (wax content). The ethanolic solvent was finally evaporated to determine the resin content (% *w/w*). These results were used for comparative purposes for the determination of the most effective method to determine the dry residue.

### 4.3. Industrial Production of Oil Extract of Propolis (OEP)

For the industrial production of oil extract of propolis, a ratio of 1-part raw propolis to 3-parts sunflower oil was used. After pressing, only the insoluble components were removed, incorporating the resin and wax into the oily matrix, according to the patented process [9], which was licensed to the ITA Brasil apiary.

### 4.4. Development of a Method to Recover the Propolis Resin from the OEP

Methanol, ethanol, isopropanol and mixtures thereof were tested to recover the resins from the oily matrix. Initially, approximately 1 g of oily propolis was weighed in triplicate and extracted with 10 mL these solvents. After mixing and filtering in a freezer, the solvents were evaporated and the dry residue was weighed. The mass of the residue and chromatograms were compared to determine the most effective solvent.

### 4.5. Total Flavonoid Content of OEP

To determine the total flavonoid content, we used the quercetin standard analytical curve method [16], but dissolved in the same solvent as the samples, ethanol: isopropanol 95:5 (EI); at concentrations of 20, 50, 100, 200, and 300 μg/mL through a serial dilution of a stock solution (1000 µg/mL) and analyzed using the same method as the samples (calibration curve). For this analysis, the samples did not require dilution; therefore, 200 μL of the samples or curve points were added to a 10 mL volumetric flask containing 5 mL of methanol. Then, 200 μL of the 5% AlCl3 reagent in distilled water was added, and the flask was made up to volume with methanol. Then, the absorption was read at 425 nm on a Thermo-Fischer Multiskan Photometer (Thermo Fisher Scientific, Waltham, MA, USA), and the results compared to those of the calibration curve.

### 4.6. Total Phenolic Contents of OEP

For total phenolic contents, we used gallic acid standard [17] dissolved in the same solvent as the samples (EI) at concentrations of 5, 10, 25, 50, and 100 μg/mL through serial dilution from a 1000 µg/mL stock solution (calibration curve) and analyzed using the method described below. The samples were further diluted 10-fold in EI before continuing the analysis. An aliquot of 0.5 mL of the diluted sample solution or calibration curve point was placed in a test tube, 2.5 mL of 10% Folin–Ciocalteu reagent in water was added, shaken, and left to react for 5 min in the dark. After 5 min, 2 mL of previously prepared 7.5% (*m*/*v*) Na_2_CO_3_ in water was added, stirred, and allowed to react for at least 2 h in the dark. After 2 h, approximately 1 mL of the supernatant was placed in a cuvette and absorbance measured at 760 nm nm on a Thermo-Fischer Multiskan Photometer. A blank sample with EI and the reagents used in the correct proportions was also measured and the results compared to those of the calibration curve.

### 4.7. Antioxidant Activity Measured by the Inactivation of the Stable Free Radical 2,2-Diphenyl-1-picrylhydrazyl (DPPH)

A 90 uM solution of 2,2-Diphenyl-1-picrylhydrazyl (DPPH) was prepared in methanol and 1 mL of this solution was added to 1 mL of the sample/standard or blank, and the volume was made up to 4 mL with methanol. The reaction remained in the dark at room temperature. Readings at a wavelength of 515 nm were taken on a Thermo-Fischer Multiskan Photometer spectrophotometer after 30 min and after 1 h. The percentile inhibition was determined according to the equation: 100 × (Absorbance of the blank − Absorbance of the sample)/Absorbance of the blank and the different % inhibition of the different concentrations of the samples or standards was used to produce a graph and thus determine the concentration that causes 50% inhibition (ED50) [18].

### 4.8. Dry Mass

Two methods were compared to determine the dry mass of propolis resin in the OEP. In the in-house method, a 1 mL aliquot of the supernatant of the extraction with EI solution was placed in a previously weighed Eppendorf flask dried in a Speed-vac, and weighed again. The difference in mass was attributed to the extracted mass (dry mass) and calculated as a percentage.

This in-house method was compared to the classic method applied to oils [11], which was not the first choice due to the use of more toxic organic solvents and the use of SepPac cartridges (silica), making it a less sustainable method. This method separates the solution into three phases: nonpolar (solvent), polar 1 (solvent), and polar 2 (solvent). Since the nonpolar phase contained the oil and residual wax, these were discarded and only the polar phases 1 and 2 were weighed. This test was performed for batches 2022, 2023 and 2024.

### 4.9. Ultra-High-Performance Liquid Chromatography with Mass Spectrometry (UHPLC-MS)

Initial sample analyses by UHPLC-MS with electrospray were performed on a UHPLC Acquity chromatograph coupled to an Acquity TQD mass spectrometer. Chromatography was performed with a C18 column. Mobile phase A (water with 0.1% formic acid) and B (methanol) were used in a gradient between 25% B and 100% B over 7 min, maintaining 100% B for 1 min, then returning to stabilize at 9.1 min to 10 min, with injection of 5 µL and column oven at 30 °C. ESI negative ion mode parameters were capillary 3.0 KV, cone 30 V, Source Temperature 150 °C and Desolvation Temperature 350 °C.

For compound quantification, the same chromatographic method was used in a Vanquish UHPLC coupled to a HRMS Orbi–Trap Exploris instrument (Thermo Fisher Scientific—Waltham, MA, USA); the MS conditions were set as default according to the LC flow used. The areas of selected peaks were compared with standard curves of caffeic, chlorogenic and p-coumaric acids to identify and quantify these components in the extract, and an internal standard (non-commercial) was used to identify the Artepillin C peak (3,5-diprenyl-4-hydroxycinnamic acid).

### 4.10. Cytotoxicity Tests

The neutral red uptake cytotoxicity assay assesses cell viability and was performed according to the Guidance document on using cytotoxicity tests to estimate starting doses for acute oral systematic toxicity tests [12]. The result obtained in the assay (concentration that inhibits 50% of cell viability—IC_50_) is used to estimate the initial lethal dose for 50% of animals (LD50) in acute systemic oral toxicity studies. Therefore, this assay allows for a reduction in the number of animals used in in vivo experiments. In BALB/c 3T3 cells (ATCC CCL-163—obtained from BCRJ—Rio de Janeiro Cell Bank, Rio de Janeiro, Brazil), Sodium Dodecyl Sulfate (SDS) was used as the positive control in the assay, as it is recognized as cytotoxic. Therefore, the test item was prepared at the highest soluble concentration according to solubility tests and tested at eight different concentrations. The cultures were examined after 48 h, and the amount of neutral red present in viable cells was determined by spectrophotometry on a Thermo-Fischer Multiskan Photometer. The positive control SDS presented an IC50 value of 62.00 μg/mL and an estimated LD50 value of 490.7 mg/kg.

### 4.11. Antimicrobial Activity

The antimicrobial activity of the ethanolic extract of green propolis and the oil extract of green propolis were performed according to the methods of the Clinical and Laboratorial Standards institute to evaluate sensitivity to antimicrobial agents [19,20,21]. In brief, both samples were serially diluted (in triplicate) and tested against *Candida albicans* ATCC1031, *Staphylococcus aureus* ATCC 6538, *Streptococcus pyogenes* ATCC 19615, *Streptococcus mutans* ATCC AU159 and *Pseudomonas aeruginosa* ATCC 13388 using the microdilution method. Nystatin and Chloramphenicol were used as control antibiotics. Mueller Hinton Agar was used for *S. aureus* and *P. aeruginosa*, RPMI1640 for *C. albicans* and BHI + cysteine for *S. pyogenes* and *S. mutans*. The plates were incubated according to the cited norms, and then TCC was added. The plates were incubated for another 3 h and the concentrations in which none of the triplicates were colored, indicated the Minimal Inhibitory Concentration. An aliquot of 10 µL was taken from these wells and inoculated in a new plate to determine the minimal bactericidal and fungicidal concentrations (MBC and MFC).

## 5. Conclusions

The direct extraction of propolis bioactive compounds by oils is a completely green process, without the use of organic solvents. During this study, we were able to establish a method to reproducibly recover the bioactive resins from OEP, so that the product could be analyzed using common, traditional methods, with already established standards. The quality of oil extract can be evaluated in the same way as ethanol extract. Adjustments of the solvents to facilitate propolis solubility are described herein, with the best results obtained using ethanol: isopropanol (95:5). The OEP has a similar overall chromatographic profile and antimicrobial activity. In addition, the results of the cytotoxicity assays indicate that it is as safe to use as the ethanolic extract. Furthermore, the total phenolic and flavonoid contents of OEP are similar to that of the ethanolic extract and meet the Brazilian Ministry of Agriculture standard for ethanolic propolis extracts and showed strong antioxidant activity. Therefore, OEP represents a promising alternative for products that currently use the ethanolic extract of propolis, without the drawbacks of the presence of alcohol in these formulations, although further validation is required.

## Figures and Tables

**Table 1:** MIC, MBC and MFC of Ethanol extract and oil extract of propolis in mg/mL.

	*Candida albicans* ATCC 1031	*Staphylococcus aureus* ATCC 6538	*Streptococcus pyogenes* ATCC 19615	*Streptococcus mutans* ATCC AU159	*Pseudomonas aeruginosa* ATCC 13388
	MIC mg/mL	MIC mg/mL	MIC mg/mL	MIC mg/mL	MIC mg/mL
EEP	2.000	0.2500	nd	0.2500	2.000
OEP	nd	0.0390	5.000	0.0024	2.500
	MFC mg/mL	MBC mg/mL	MBC mg/mL	MBC mg/mL	MBC mg/mL
EEP	2.000	0.250	nd	0.2500	2.000
OEP	nd	0.039	5.000	0.31	nd

nd. Not determined.

**Table 2:** Concentration in µg/mL of: chlorogenic acid, caffeic acid, p-coumaric acid, quercetin (quantified by comparison with the calibration curve of the standards) and Artepillin-C (identified by comparison of Rt and *m*/*z* of donated isolated compound and quantified using the caffeic acid standard curve).

Compound	*m*/*z*	Rt min	EEP Mean µg/mL	OEP 2022 Mean µg/mL	OEP 2023 Mean µg/mL	OEP 2024 Mean µg/mL
Chlorogenic acid	353.23	1.5	9.15	0.02	0.02	0.02
Caffeic acid	179.08	1.9	0.95	0.05	0.03	0.04
p-Coumaric acid	163.12	2.5	13.65	7.94	7.60	8.58
Quercetin	301.33	4.2	0.87	0.12	0.12	0.12
Artepillin-C	299.18	6.5	102.40	287.57	274.36	256.07

**Table 3:** Total flavonoid content expressed as mean and standard deviation (SD) in samples from OEP batches 2022, 2023, 2024 and sunflower oil.

Sample	Mean%	SD%
2022	0.55	0.06
2023	0.44	0.02
2024	0.45	0.04
oil	0.02	0.01

**Table 4:** Total phenol content (in % *m*/*m*) presented as mean and standard deviation in samples from OEP batches of 2022, 2023, 2024 and sunflower oil.

Sample	Mean%	SD%
2022	2.36	0.233
2023	1.70	0.188
2024	2.05	0.112
oil	0.17	0.022

**Table 5:** ED50 determined by the DPPH method (ug/mL) for batches OEP 2022, 2023, 2024, reading after 30 min and 1 h.

Sample	Mean After 30 min	Mean After 1 h
2022	21.83	18.98
2023	27.11	24.88
2024	29.84	30.21
Average 3 batches	26.26	24.69

**Table 6:** Dry mass (in % *m*/*m*) presented for samples from OEP batches of 2022, 2023, 2024 using the in-house method.

Sample	Mean% *m*/*m*	SD%
2022	20.48	3.87
2023	19.89	3.73
2024	19.68	3.55

## Data Availability

Any further data may be obtained from the authors upon reasonable request.

## Supplementary Materials

The following supporting information can be downloaded at: https://www.mdpi.com/article/10.3390/molecules31081234/s1. Table S1. Areas of ions extracted from the UHPLC-MS chromatogram of the extracts using 5% isopropanol in ethanol (EI), ethanol and methanol of OEP; Table S2. Dry mass (in % m/m) presented for samples from EOP batches of 2022, 2023, 2024 divided into Polar 1, Polar 2 and summed phases.

## Abbreviations

The following abbreviations are used in this manuscript:

OEP	Oil extract of propolis
EEP	Ethanol extract of propolis
EI	Ethanol: isopropanol
XIC	Extracted Ion Chromatogram
