# Supplementary material for: Oil Extract of Green Brazilian Propolis, Antioxidant Activity, Safety and Quality Control
Source: Molecules. 2026 Apr 8;31(8):1234. doi: 10.3390/molecules31081234 (PMC13118830; doi:10.3390/molecules31081234)
Supplement: Supplementary file 1 [file molecules-31-01234-s001.zip › molecules-4219817-supplementary.pdf]

Supplementary tables.

Table S1. Areas of ions extracted from the UHPLC-MS chromatogram of the extracts using 5% isopropanol in ethanol (EI), ethanol and methanol of OEP.

Solvent	Peak area *m/z* 163 (p-coumaric acid)	Peak area *m/z* 299 (artepillinC)
5% isopropanol / ethanol	625.612	6.228.467
ethanol	838.330	2.955.020
methanol	837.102	2.904.495

Table S2. Dry mass (in % m/m) presented for samples from EOP batches of 2022, 2023, 2024 divided into Polar 1, Polar 2 and summed phases

Sample	Polar 1(%)	Polar 2 (%)	Sum 1+2 (%)
2022	4.47	46.39	50.86
2023	5.57	35.42	41.00
2024	6.67	35.78	42.45
